# Sleep disordered breathing from preschool to early adult age and its neurocognitive complications: A preliminary report

**DOI:** 10.5935/1984-0063.20200098

**Published:** 2021

**Authors:** Kyriaki Astara, Dimitra Siachpazidou, George D Vavougios, Dimitrios Ragias, Konstantina Vatzia, Georgia Rapti, Emmanouil Alexopoulos, Konstantinos I Gourgoulianis, Georgia Xiromerisiou

**Affiliations:** 1 School of Medicine, University of Thessaly - Larissa - Thessaly - Greece.; 2 University of Thessaly, Department of Respiratory Medicine, Faculty of Medicine, School of Health Sciences - Larissa - Thessaly - Greece.; 3 Athens Naval Hospital, Department of Neurology - Athens - Athens - Greece.; 4 School of Medicine and Larissa University Hospital, Sleep Disorders Laboratory, University of Thessaly - Larissa - Thessaly - Greece.; 5 University of Thessaly, University Hospital of Larissa, Department of Neurology - Larissa - Thessaly - Greece.

**Keywords:** Sleep Apnea Syndromes, Cognitive Dysfunction, Adolescent, C-Reactive Protein

## Abstract

**Objective:**

The onset and development of sleep disordered breathing (SDB) remains unclear in an age - dependent manner. Despite treatment, persistent symptoms such as snoring and excessive daytime sleepiness, as well as cognitive impairment may be present. The aim of the research was to determine the prevalence of residual symptoms of SDB in adolescence and early adulthood, the predisposing factors and its neurocognitive complications.

**Methods:**

In the present pilot study-cohort, a questionnaire was utilized to 154 people (average age: 17.9 ± 3), who as children (mean age: 5.3 ± 1.4) had AHI ≥2.5 episodes/h. They were divided into two groups based on AHI = 5 episodes/h. Depending on the results, they were invited to undergo a repeated polysomnography (PSG) and complete the Montreal Cognitive Assessment (MoCA) test. Statistical analysis was made with IBM SPSS software.

**Results:**

Out of the total, 35.7% claimed to still snore. AHI was negatively correlated to the severity of residual symptoms (Mann-Witney U test, p <0.005). According to repeated PSGs, 9/17 met the criteria for OSAS, while high BMI was associated with the severity of new AHI (chi squared test, p<0.005). Additionally, 7/16 scored below the MoCA baseline (<26/30). The characteristics of cognitive declines were mapped, with most prominent having been visuospatial, short - term memory and naming/language deficits.

**Discussion:**

A significant percentage of children with sleep breathing disorder present with residual symptoms during their transition to early adulthood, as well as undiagnosed neurocognitive complications. Clinicians suspicion for the underlying neurocognitive complications is required, even in young adults, while guidelines on monitoring pediatric OSAS patients after treatment should be addressed.

## INTRODUCTION

Sleep disordered breathing (SDB) encompasses a wide spectrum of abnormal breathing during sleep including primary snoring, upper airway resistance syndrome, obstructive hypoventilation syndrome and obstructive sleep apnea-hypopnea syndrome^1^. From all the SDB syndromes, the most significant in terms of severity, prevalence and underdiagnosis is the obstructive sleep apnea syndrome (OSAS)^2^. A biphasic peak of the prevalence of OSAS occurs between 2 and 8 years, as well as during adolescence. The most common causes are adenotonsillar hypertrophy and obesity, which both seem to share a common inflammatory background^3,4^. The significance of the underlying inflammatory processes is mostly prevalent through the therapeutic results of anti-inflammatory treatments in mild cases, which include the reversal of symptoms, PSG values and complications^5^.

It has been reported that OSAS, under certain circumstances like obesity^6^ and male gender^7^, tends to persist after treatment, regardless of the therapeutic approach^8,9^. Despite the high success rates of adenotonsillectomy in children^10^ and the proper application of CPAP in adults^11^, patients exhibit either snoring or residual excessive daytime sleepiness (REDS). Although it was considered as a benign symptom, these obstructive symptoms might conceal more serious underlying conditions^12^.

REDS has been associated with cognitive impairment, especially in adults^13^. Despite the proper appliance of continuous positive airway pressure (CPAP) - the gold standard of treatment in adults and an alternative approach in children - and the patients' compliance, REDS usually persists, counteracting the benefits of CPAP in cognitive health^11,14^. REDS has been partly attributed to mechanisms that activate neurodegenerative processes, such as sleep disturbances, due to cortical arousals, as well as to intermittent hypoxia^15^. However, the mechanisms that occur on a cellular level and on the level of blood brain barrier (BBB) have not been established yet^16^. Even though cognitive impairment as a correlate of aging affects adults^17^, it has been described in the pediatric population as well^18^. Children seem to complain less about REDS^19^, probably due to the normal sleep architecture. However, subcortical arousals, which are not detected on PSGs, occur^20^ contributing to rather neurobehavioral complications^21^, alongside with multifocal domain dysfunction like memory problems, attention impairments, learning disabilities and lower intelligence scores^22^. To date, the interrelationship between OSAS and cognitive/behavioral effects remains to be fully elucidated, in order to acknowledge the neurodevelopment sequelae in children.

The primary aim of this study was to estimate the prevalence of the most prominent symptoms of residual OSAS in the context of the transitional period of adolescence, their predisposing factors as well as to define the complications through the levels of C - reactive protein (CRP) and a cognitive assessment.

## MATERIAL AND METHODS

### Questionnaire

All the children that had had a sleep study from January 2001 up to December 2010 were enrolled. The children were selected from the database of the sleep laboratory of pulmonology clinic of the University Hospital of Larissa. Inclusion criteria were a previous diagnosis of OSAS via PSG with AHI≥2.5. Exclusion criteria concerned only cognitive assessment, for individuals with mental incapabilities due to underlying conditions (e.g., Prader-Willi syndrome). Two groups were formed based on the AHI of their first PSG. The first group contained all the patients with AHI range 2.5-4.9, whereas the second the ones with AHI≥5 episodes/h. They were approached via the telephone by a group of researchers to complete a questionnaire, which was a variation of the Berlin questionnaire (Supplementary Table 1). The respondents were asked about their anthropometric features, like weight and height, their age, educational level, as well as whether they still snore. To whomever responded positively, additional descriptive questions about this symptom were asked. The intensity and frequency of snoring, whether it has become annoying to third parties, possible episodes of apnea, sleep exhaustion after sleep and during the day, and the possibility of falling asleep while driving, with the corresponding frequency, were evaluated. In addition, the treatment method that was followed and if there has been a treatment for arterial hypertension, were recorded. Intensity was calibrated as "slightly more intense than breathing", "as intense as speech", "more intense than speech" and "very loud, it can be heard from the next room". The episodes of apnea, the frequency of snoring, the exhaustion after sleep and during the day, and the frequency of falling asleep on the wheel, were calibrated as "almost every day", "1-2 times a week", "3-4 times per week","1-2 times a month" and "never or almost never". The recipients of the questionnaires were able to note comments and observations concerning the symptoms in the middle of the questionnaire, and more general ones at the end of the questionnaire. For every patient, BMI was calculated and recorded. For patients under 21 years old, BMI was calculated in the same way as for adults, but then compared with the percentile for children of the same sex and age.

### Polysomnography

According to the questionnaire's results, only the respondents who claimed to still snore were invited to undergo a repetitive PSG at the Sleep Laboratory of Respiratory Medicine Department of University of Thessaly, for the evaluation of their symptoms. Electrocardiograph, electrooculography of both eyes and electroencephalograph were obtained during PSG. The flow of air through the nose and mouth was evaluated using a special thermistor and thoracic and abdominal movements were calculated by inductive plethysmography. Saturation of haemoglobin (SaO_2_) was recorded by a pulse oximeter and snoring by a microphone application. All sessions were performed under the same conditions in the facilities of Sleep Laboratory with environmental temperature at 24±1 ºC, humidity 47±2% and quite level <30dB.

**Figure 1:**
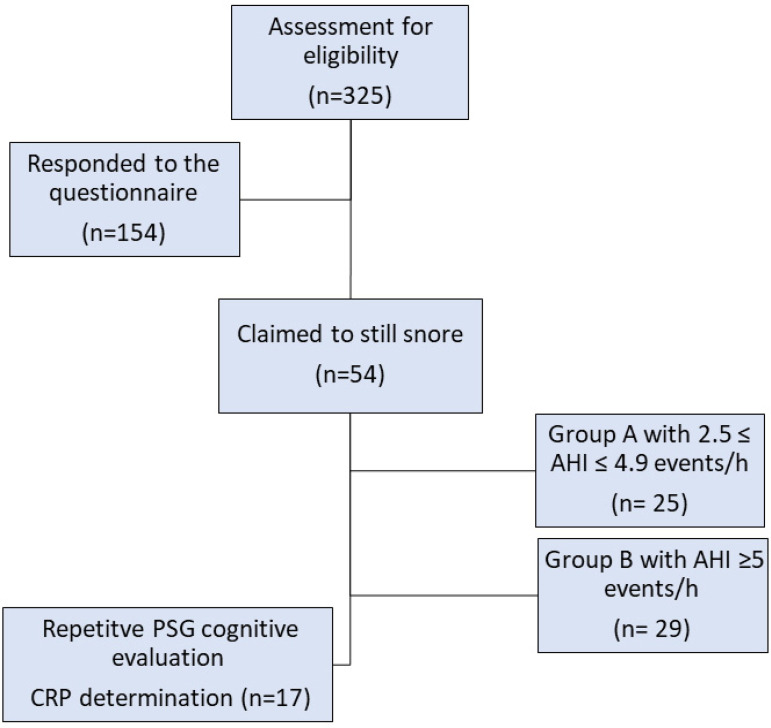
Flow chart of the study phases.

Upon their arrival, participants and their legislated guardians submitted a written consent, after a trained medical doctor explained the protocol, related procedures concerning PSG, cognitive evaluation and blood sampling, as well as the purpose of the study. The participants were able to express any concerns or queries, regarding the research, while their anonymity and confidentiality were ensured. A special neuropsychiatric test to assess cognitive health was performed at night before sleep and blood sampling to determine CRP, the next morning. For the cognitive evaluation, the Montreal Cognitive Assessment (MoCA) test was utilized (Figure 1). The study was conducted according to the Helsinki declaration for use in Human subjects (No. 25731/16-07-2020, Scientific Council of University Hospital of Larissa, Greece).

For serologic calculation of CRP levels, venous blood was taken after fasting in the morning from a vein of the arm. At least 5ml of blood, without anticoagulant, was received. It was then be centrifuged and the serum was placed in the freezer at -70˚C. When all the blood samples were collected, analyses were performed at the same time after a single batch defrost. CRP levels were quantified by a high-sensitivity immunonephelometric method with a lowest detection limit of 0.0175mg/dl.

### Statistical analysis

Data are presented as the means ± standard deviation (SD) where applicable. Data normality was assessed with the Kolmogorov-Smirnov test. Comparisons between the two groups were performed via Mann-Whitney U according to variable distribution. Data from PSGs were analyzed as categorical variables, via chi-squared test, for the detection of possible risk factors. Data analysis was performed using the IBM SPSS Statistics software for Windows, version 25 (IBM Corp., Armonk, NY, USA) and Microsoft Excel Software for Windows, version 16 (Microsoft Corp., Redmond, Washington, USA). For all tests, a *p*-value<0.05 was considered to indicate a statistically significant difference. Guttman's Lambda 4 coefficient was chosen as the index of internal consistency.

## RESULTS

### Questionnaire

In total, 325 people were approached via telephone (AHI range: 2.5-62.1). A total of 154 were included in the study, having responded and completed a questionnaire (response rate: 40.2%). The internal consistency reliability of the questionnaire was approximately 0.8. Among them, men were 62% and the rest were women. The age range was from 12 to 25 years (mean age: 17.9±3), with the half of them being under eighteen years old. Regarding the educational level of the respondents, 53.9% of the participants was in secondary education, 44.8% in third and in primary the 1%. Concerning residual symptoms, approximately one out of third (n=54 subjects, 35.7%) claimed that they still snore. Out of this percentage, 27.7% characterized its intensity as "slightly more intense than breathing", 44.4% as "as intense as speech", 18.5% as "more intense than speech" and 9.30% as "very loud, it can be heard from the next room". Regarding the frequency of snoring and exhaustion after sleep and during the day, the results are shown in Table 1. According to Table 1, the symptoms tend to concern the majority participants on a weekly basis. The 63% claimed to present these obstructive episodes, while the 54% complained about waking up and the 58% feeling exhausted during the whole day.

**Table 1.** Frequency of snoring and exhaustion after sleep and during the day.

Frequency	How often do you snore?	How often do you feel exhausted after sleep?	How often do you feel exhausted during the day?
Never/almost never, %	0	31.48	24.07
1-2 times/month, %	37.00	14.81	12.96
1-2 times/week, %	26.00	20.37	16.67
3-4 times/week, %	16.67	25.93	30.00
Almost everyday, %	20.37	7.41	16.67

In the question whether their snoring has become annoying to others, 61.5% responded negatively. In case of episodes of apnea, 19.2% (10 in absolute number) replied that they present "1-2 times a month" (n=5), "1-2 times a week" (n=2), "34 times a week" (n=1) and "almost every day" (n=2). Of all the snorers, only one reported that he falls asleep while driving, at a frequency of "1-2 times a week".

All the respondents were divided in two groups, according to the former AHI they had on their previous diagnosis of OSAS. The formation of the two groups based on AHI aimed to examine the possibility of an interrelationship between AHI and the severity of residual symptoms. The first group contained the subjects with AHI: 2.5-4.9 episodes/h whereas the second, the ones with AHI≥5 episodes/h. Table 2 demonstrates the results of the questionnaire concerning the two groups in comparison, separately.

**Table 2.** Results of each group separately. Data are expressed as mean ± standard deviation or percentages. The p-value refers to the Mann-Whitney U test for comparing variables between groups. Significant differences concerning the severity of symptoms and AHI, were observed (severe obstruction: p<0.001, exhaustion after sleep: p<0.05, daytime exhaustion: p<0.05).

Results	Total n=54	Group A (2.5 ≤ AHI ≤ 4.9) n=25	Group B (AHI ≥ 5) n=29	p-value
Age, years	18.9±3.1	19.7±3.3	18.3±2.8	-
Gender (male, %)	72	68	76	-
Severe obstruction	27.8	32.0	24.1	<0.001
Frequency in weekly basis…				
…snore, %	63.0	68.0	55.1	NS
…exhaustion after sleep, %	53.7	56.0	51.6	<0.05
…daytime exhaustion, %	63.0	76.0	55.1	<0.05
Abnormal BMI…				
…underweight, %	0	0	0	NS
…overweight, %	14.8	8.0	28.0	NS
…obese, %	16.7	16.0	21.0	NS
Treatment…				
…none, %	29.6	40.0	21.0	NS
…surgery, %	53.7	44.0	62.1	NS
…conservative, %	7.4	4.0	10.3	NS
…combination, %	9.3	12.0	6.9	NS

Polysomnography studies

Since none of the measurements displayed parametric distribution based on the Kolmogorov-Smirnov test, non-parametric tests (i.e., Mann-Whitney U and chi-squared test) were used. The comparison between the two groups displayed an increase in the severity of most the symptoms (intensity of snore as well as frequency of exhaustion after sleep and during the day) in the lower AHI group (group A) (Mann-Whitney U test, *p*<0.001 and *p*<0.005, respectively).

**Figure 2:**
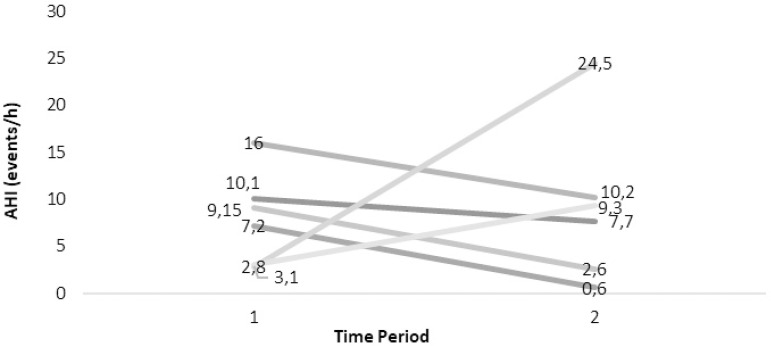
Natural History of SDB of the cases undergone two PSGs. Dark grey lines indicate residual OSAS (AHI=5).

**Figure 3:**
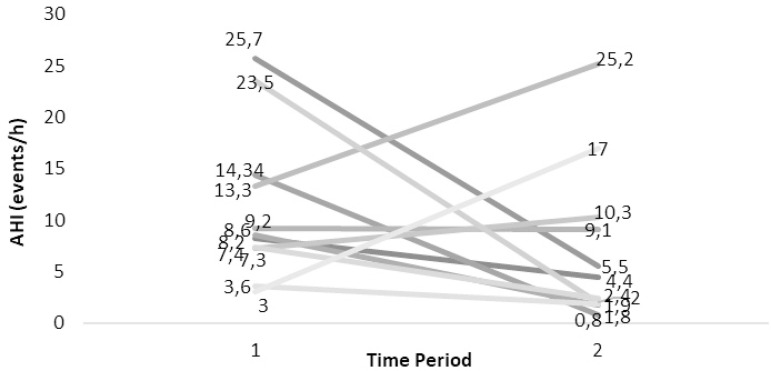
History after Treatment of SDB of the cases undergone two PSGs. Dark grey lines indicate residual OSAS (AHI=5).

All subjects that responded positively about snoring were invited to undergo a repetitive PSG, to further evaluate their claimed obstructive phenomena. From the sample that claimed to still snore (n=54), 17 individuals agreed and underwent a sleep study at the Sleep Laboratory. Figure 2 and Figure 3 demonstrate the evolution of OSAS according to the AHIs from their first and the repetitive PSG. 8/17 cover the adult criteria for OSAS (AHI≥5). They were divided in two groups, the one in which no treatment was followed during the period of the first PSG and the other that either surgery or a conservative approach was chosen.

The past and recent anthropometrics and polysomnographic features, along with C-RP levels, are shown in Supplementary Table 2 and Supplementary Table 3. Table 3 demonstrates the results of the neurocognitive assessment in relation to age and BMI. The 71% were male, the 44% has abnormally high BMI and 44% (7/16) scored below the baseline (<26/30) of MoCA test (sensitivity: 90% and 100% adjusted in mild AD group, and specificity: 87%). All CRP measurements were within normal range (Supplementary Table 3). AHI severity was associated with several risk factors (Supplementary Table 3), but only BMI yielded a significant correlation (chi-squared test, *p*<0.005).

**Table 3.** The MoCA results corresponded with the age, BMI and the treatment approach of each individual. One was excluded due to Prader-Willi syndrome.

	MoCA (score)		Age (years)		BMI (kg/m2)		Treatment		AHI (events/h)	
	<26	≥26	<17	≥17	<25	≥25	Yes	No	<5	≥5
p-value	NS	NS	NS	NS	NS	NS	NS	NS	NS	NS

Note: AHI = Apnea-hyponea index; BMI = Body mass index; MoCA = Montreal Cognitive Assessment; Treatment was defined as dichotomous variable: Treatment = 0 corresponds to the absence of therapy, Treatment = 1 to the presence of any.

**Figure 4:**
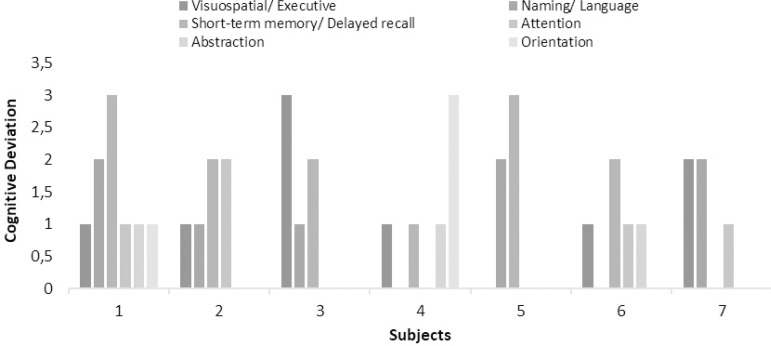
Clustered column chart with cases that scored below 26/30 in MoCA per domain.

As all neurocognitive tests, MoCA contains clusters of tasks that represent each cognitive domain. The 7/16 cases that scored below the baseline of MoCA, had their scores analyzed in each domain separately. Subsequently, the results were converted to detect the cognitive domains declined by recording the deviation or otherwise the score of each for every wrong answer and are presented in Figure 4.

**Figure 5:**
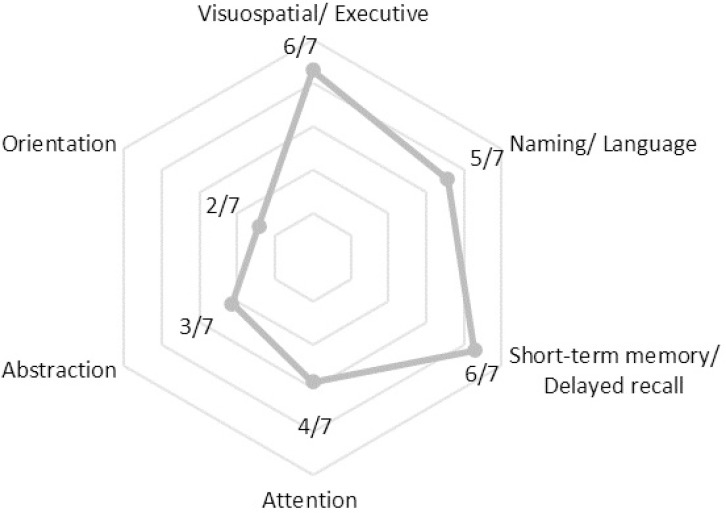
Radar plot with the features of cognitive performance defined by the domains of decline.

The characteristics of cognitive performance and the deviations from the mean are displayed in Figure 5. Thus, there are candidate cognitive decline phenotypes defined by the domains.

## DISCUSSION

Our results have shown that during the transitional stage of adolescence, a considerable proportion exhibits persistent symptoms of SDB, like snore and REDS, as well as undiagnosed neurocognitive deficits. It is unclear whether OSAS under certain circumstances as a continuum throughout the transition from childhood to adulthood or two different age-defined phenotypes. Despite the evidence that supports different pathophysiology between pediatric and adult OSAS^23^, several studies have addressed the natural history of OSAS through ages^24,25^ as well as the long-term effects^26^ with the factors that contribute to this persistence remaining to be elucidated.

A significant percentage claimed to still snore on a weekly basis, in the questionnaire that was utilized as a screening method. However, the risk of underestimation is substantial as the symptoms are underreported by patients while they tend to elude clinicians' attention^27^. The results from the comparison between the two groups showed the one with the lower AHI (2.5-4.9 episodes/h) scored higher in terms of severity of the residual symptoms, compared to the group with AHI≥5 episodes/h. It was attributed to the differences of the treatment method the two groups followed. According to the data, the respondents with higher AHI followed a more aggressive treatment, mainly surgery alone or in combination with a conservative approach (mainly intranasal corticosteroids). On the other hand, the majority of the group with lower AHI recorded none or merely conservative treatment approaches.

The therapeutic efficacy of intranasal corticosteroids possibly applies only in mild pediatric cases of OSAS and is short-term^28^ as their long-term efficacy has not been established yet^29^. Therefore, the suboptimal treatment of OSAS in childhood is likely to be responsible for its persistence. Additionally, the age dependence of the physiology of sleep results in differences in clinical manifestation between pediatric and adult onset OSAS, extending not only to clinical phenotypes and PSG findings, but also to the evaluation of OSAS' severity^30^. These age-dependent disparities may result in both underdiagnosis of pediatric OSAS and its inappropriate therapy^31^.

Concerning the individuals with persisting SDB, our data shows that almost half were diagnosed with residual OSAS. According to the results and relevant literature^32^, high BMI occurred as predicting factor. Weight gain and sleep apnea seem to be interrelated. Sleep apnea may lead to weight gain due to the dysregulation of hormones concerning appetite and through the sedentary lifestyle daytime sleepiness enhances^33^. On the other hand, weight gain may worsen apneic episodes, as the extra adiposity is deposited in the neck area. Facial obesity, alongside with other craniofacial abnormalities and increased volume of intraluminal soft tissue structures, like tongue and soft palate, have been associated with the upper airway (UA) collapsibility^34,35^. Neck circumference, especially when corrected for height, has been found to serve as an additional clinical predictor of OSAS^36^. The increase of neck circumference probably worsens OSAS, by attenuating a protective reflex by UA mechanoreceptors, which prevent its collapse by enhancing the activity of UA dilators^37^. This reflex re-establishes ventilation in an alternative to arousal manner, resulting in less cortical arousals and hence sleep fragmentation.

Despite being reported as a risk factor^38^, male gender was not associated with an increased risk of residual OSAS in the present study. This could be possibly attributed to the small sample as well as the onset of OSAS in childhood. Gender differences in the prevalence of OSAS occur from adolescence and henceforth, due to mainly anatomical differentiations in the UA^39^, whereas during childhood, boys and girls demonstrate similar OSAS severity, indicating different pathophysiology^40^. In fact, adenotonsillar hypertrophy is the main cause of OSAS in prepubertal children, regardless of the gender^41^. Since the participants enrolled and re-diagnosed with OSAS, had their onset during childhood, sex was unlikely to be associated with the progress of SDB. Therefore, the factors that determine the presence of residual OSAS might be regardless of the therapy.

Due to its multifactorial origin, OSAS has reciprocally been linked to inflammation and therefore complications. Inflammation processes are assessed through elevated plasma proteins, such as CRP, which could serve as risk factor^42^. In the present study, CRP levels were normal in all individuals that underwent a repetitive PSG and were diagnosed with SDB. Even though some studies have correlated CRP levels with OSAS severity in adult patients^43^, the results remain controversial, especially in children^44^. Due to the highly variable causality of elevated CRP levels, even marginal elevations are difficult to interpret, especially when not combined with other tests^45^. Elevated levels would require clinical correlation, as the most common comorbidities of OSAS, like obesity and hypertension, could independently make inflammation processes become more explicit^46^.

Despite the lack of association of OSAS with inflammatory processes based on CRP, MoCA test results supported the detrimental effect of the syndrome on cognitive function. In fact, almost half of the respondents scored below baseline (<26/30), indicating neurocognitive deficits. The MoCA test seems to be superior in the detection of mild cognitive impairment (MCI) than other tools (e.g., mini-mental state examination (MMSE) with sensitivity: 18% and 78% when adjusted, and specificity: 100%)^47^ even in the early stages of decline^48^. The MoCA results were expected to be underestimated, given the ample cognitive reserves relatively young and educated individuals, as our participants, expected to have^49^. Nevertheless, adjusted to age cut-offs are not available, yet^50^. Furthermore, it allows the allocation of cognitive decline to a cognitive domain, allowing the extrapolation of a cognitive decline phenotype. Visuospatial, short-term memory and naming/language deficits were the most prevalent in the study population. In a meta-review study, most of the above domains were linked to hypoxia-hypercapnia and EDS observed in OSAS, with the visuospatial deficits being unique feature^51^. In adolescents, maturation during sleep begins at the back of the brain, where visual and spatial perception are performed, and progresses forward^52^. This fundamental process is hindered by sleep fragmentation in OSAS, with its implications being radiated to the relevant cognitive domain. Endothelial dysfunction and cerebrovascular disease have, also, been addressed as possible mechanisms^53^, but data are mixed concerning visual perception^54^.

The characteristics of declining cognitive performance were approached as ordinal variables, acknowledging, and proposing cognitive-impairment phenotypes to enhance clinical suspicion. Although cognitive deficits are not expected in younger adults, they seem to be present and alarmingly underrecognized. The necessity to screen for these deficits emerges in the context of standardized assessment for larger scale research upon scrutinizing causality. Since these are preliminary results, the official diagnosis of MCI cannot be made in young adults, until the cause and effect link between MCI and OSAS is documented. The way the complications of the syndrome undermine the quality of life has not been fully unveiled, it could be suggested that they predispose for comorbidities^55^ and increased risk for accidents^56^.

### Limitations

Our research had some limitations. The questionnaire utilized was small, as it was modified for telephone approach. The systematic error due to the researchers' bias in interpreting the symptoms was unavoidable. Additionally, several questionnaires are available for the screening of sleep apnea, but none has been verified because of low precision, resulting in inadequate validity^57^. The results about the diagnostic accuracy of Berlin questionnaire are generally mixed and satisfying in discriminating severe cases of OSAS^58^. As mentioned above, the risk of underestimating the clinical manifestation via the questionnaire exists signifying that, ideally, all the respondents should undergo a PSG. Regarding the respondents for a repetitive PSG, only a few were enrolled, leading to a small sample size and inevitable statistical bias in our results. Lastly, the individuals were not tested for various causes that could overshadow the mental capabilities. Attention deficit/hyperactivity disorder (ADHD) is one of the most commonly diagnosed behavioral health disorders, principally diagnosed in childhood but tends to evade the clinicians' attention in adulthood^59^. ADHD is not only a rather heterogeneous disorder with several subtypes, but also demonstrates developmental changes through age^60^. Hence, the possibility of coexisting conditions acting as a coverage of cognitive abilities could not be excluded. However, the study design was not specifically directed in revealing cognitive deficits, but rather to map their existence and provide the rationale for closer scrutiny, as a pilot study.

## CONCLUSION

In conclusion, a significant proportion was presented with residual symptoms and undiagnosed neurocognitive impairment. Clinical suspicion may be necessary even in mild cases with young adults. Therefore, the necessity for larger studies emerges, to identify residual SDB and the underlying features and mechanisms towards cognitive complications, as well as to modify current guidelines for monitoring children and adolescents, especially obese individuals, after the treatment of OSAS.

## Appendix

**Supplementary Table 1.** Questionnaire for OSAS - Variation of the Berlin Questionnaire.

Questionnaire for Obstructive Sleep Apnea Syndrome (OSAS)
Variation of the Berlin – Questionnaire
**1.Gender ……………….. Age ………………..……… Weight …………….. Height ……………….. Education Level ……………………..**
**2. Do you snore?**
• Yes
• No
• Do not Know
If participant answered YES proceed to questions 3 – 10. Otherwise, procced to question 11:
**3. How would you describe your snore?**
• Slightly more intense than breathing
• As Intense as speech
• More intense than speech
• Very loud It can be heard from the next room
**4. How often do you snore?**
• Almost every day
• 1 -2 times a week
• 3-4 times per week
• 1-2 times a month
• Never or almost never
**5. Has your snore become annoying to third parties?**
• Yes
• No
**6. Has anyone noticed that you stop breathing during sleep?**
• Almost every day
• 1 -2 times a week
• 3-4 times per week
• 1-2 times a month
• Never or almost never
**7. How often do you feel exhausted after sleep?**
• Almost every day
• 1 -2 times a week
• 3-4 times per week
• 1-2 times a month
• Never or almost never
**8. How often you feel tired during the day?**
• Almost every day
• 1 -2 times a week
• 3-4 times per week
• 1-2 times a month
• Never or almost never
**9. Have you ever fallen asleep while driving?**
• Yes
• No
**10. If participant answered YES, how often does this happen?**
• Almost every day
• 1 -2 times a week
• 3-4 times per week
• 1-2 times a month
• Never or almost never
**11. Do you have high arterial blood pressure?**
• Yes
• No
• Do not Know
**12. Treatment method during the first PSG: ……………………………………………….**
**13. BMI = ……………………………………………….**

(Note: for patients under 21 years old, BMI should be compared with the percentile for children of the same sex and age)

**Supplementary Table 2.** Former Anthropometric and Polysomnographic Features.

Gender	Age	BMI	AHI	Desaturation Index	Nadir O2	<95 min	<90 min	C- RP (mg/dl)
M	3	0	9.2	9.80	84.00	77.5	2.5	1.000
M	4	0	10.1	11.00	77.00	19.5	0.0	0.090
M	4	0	7.2	7.20	90.00	13.0	0.0	0.090
F	4	1	16	16.00	79.00	349.0	87.5	0.090
F	4	1	8.2	11.10	78.00	18.0	5.5	3.000
M	5	0	9.15	8.70	85.00	13.0	1.0	1.700
F	5	1	25.7	24.80	70.00	80.0	21.0	1.000
M	5	0	8.6	6.70	88.00	10.0	0.5	0.090
M	5	1	13.3	37.20	71.00	48.0	1.0	0.090
M	5	1	7.3	8.50	90.00	15.0	0.0	0.100
M	5	0	7.4	7.80	90.00	8.0	0.0	0.100
M	6	0	23.5	28.50	81.00	63.0	8.0	0.090
M	7	0	3.1	2.80	90.00	3.0	0.0	1.000
M	8	0	14.34	13.10	83.00	23.,0	2.5	0.090
F	8	0	3.6	3.40	90.00	6.0	0.0	0.870
M	8	1	3	2.80	91.00	1.0	0.0	0.000
F	11	0	2.8	6.60	91.00	16.0	0.0	3.000

BMI = 0 corresponds to normal, BMI = 1 to obese (all categories included). M = male gender, F = female gender.

**Supplementary Table 3.** New Anthropometric and Polysomnographic Features.

Gender	Age	BMI	AHI	Desaturation Index	Nadir O2	<95 min	<90 min	C- RP (mg/dL)
M	14	1	25.2	21.9	68	32.4	1.6	0.22
M	16	0	2.4	3.2	94	0.2	0	0.01
M	17	0	7.7	5.8	87	8.9	0.7	0.1
F	17	1	10.2	4.7	88	2.5	0.2	excluded
F	17	0	4.4	2.6	89	0.3	0.1	0.05
M	17	0	1.8	0.2	95	0	0	0.11
F	17	0	10.3	11.2	80	26.1	18.1	0.02
M	17	0	2	3.2	92	35.6	0.1	0.05
M	18	1	0.6	1.3	95	5.7	0	0.06
M	18	0	5.5	1.7	93	0.8	0	0.48
M	19	1	9.3	10.9	91	24.1	0	0.04
M	20	0	2.6	1.5	95	0.1	0.1	0.05
M	20	0	24.5	25.9	80	285.7	24.2	0.02
M	20	1	9.1	10.1	88	250.2	2.2	0.11
F	20	0	1.9	2.2	86	1.1	0.4	0.06
M	22	1	17	18.2	89	292.3	8.6	0.38
F	24	0	0.8	0	95	22.4	0	0.1

BMI = 0 corresponds to normal, BMI = 1 to obese (all categories included). M = male gender, F = female gender significant correlation observed only between AHI – BMI (Chi-square test, p < 0.005).
